# Suboptimal Light Conditions Influence Source-Sink Metabolism during Flowering

**DOI:** 10.3389/fpls.2016.00249

**Published:** 2016-03-03

**Authors:** Annelies Christiaens, Ellen De Keyser, Els Pauwels, Jan De Riek, Bruno Gobin, Marie-Christine Van Labeke

**Affiliations:** ^1^Department of Plant Production, Faculty of Bioscience Engineering, Ghent UniversityGhent, Belgium; ^2^PCS Ornamental Plant ResearchDestelbergen, Belgium; ^3^Plant Sciences Unit, Institute for Agricultural and Fisheries ResearchMelle, Belgium

**Keywords:** source-sink, invertase, sucrose synthase, supplemental light, *Rhododendron simsii*, azalea, flowering

## Abstract

Reliance on carbohydrates during flower forcing was investigated in one early and one late flowering cultivar of azalea (*Rhododendron simsii* hybrids). Carbohydrate accumulation, invertase activity, and expression of a purported sucrose synthase gene (*RsSUS*) was monitored during flower forcing under suboptimal (natural) and optimal (supplemental light) light conditions, after a cold treatment (7°C + dark) to break flower bud dormancy. Post-production sucrose metabolism and flowering quality was also assessed. Glucose and fructose concentrations and invertase activity increased in petals during flowering, while sucrose decreased. In suboptimal light conditions *RsSUS* expression in leaves increased as compared to optimal light conditions, indicating that plants in suboptimal light conditions have a strong demand for carbohydrates. However, carbohydrates in leaves were markedly lower in suboptimal light conditions compared to optimal light conditions. This resulted in poor flowering of plants in suboptimal light conditions. Post-production flowering relied on the stored leaf carbon, which could be accumulated under optimal light conditions in the greenhouse. These results show that flower opening in azalea relies on carbohydrates imported from leaves and is source-limiting under suboptimal light conditions.

## Introduction

Azalea hybrids in the genus *Rhododendron* are well known for their beautiful flowers. Complete flower opening is dependent upon petal growth, which in turn requires cell division and cell expansion. Cell division in petals is the primary mechanism during the first stages of petal growth, but cell expansion becomes the dominant process responsible for full flower opening (Reale et al., 2002). Cell expansion is a combined process of cell wall weakening, carbohydrate allocation and water uptake. Expression of an expansin gene occurs during flower opening in wintersweet (*Chimonanthus praecox*) and other plant genera (Ma et al., 2012). Expansins are extracellular proteins involved in cell wall modifications that contribute to cell expansion. In rose petals, large amounts of soluble carbohydrates are known to accumulate, especially in vacuoles (Yamada et al., 2009). Carbohydrate flux into petal cells is necessary for biosynthesis and maintenance respiration, but serves also as osmoticum. Carbohydrate accumulation lowers the osmotic water potential in petal cells and promotes water influx, thus driving cell expansion (Tarpley and Sassenrath, 2006).

Sucrose is the main sugar that moves through the phloem from leaves (source) to provide soluble carbohydrates to developing flowers (sink). This transport is driven by a pressure difference between sources and sinks (Münch, 1930). Hence, sucrose metabolism is a key factor in flowering, as sucrose must be hydrolyzed to continue phloem unloading in the sinks and maintain phloem transport from source to sink. Key enzymes in sucrose metabolism are SPS, SUS, and invertases. *SPS* genes are mostly expressed in photosynthetic tissues, but SPS activity has also been shown to play a role in flower tissue of orchids and rose, where it was associated with increased sucrose content in the petals (Li et al., 2003; Kumar et al., 2007). SUS catalyzes the reversible reaction that splits sucrose into fructose and UPD-glucose. SUS activity in sink organs is correlated with sucrose unloading and sink strength in tomato fruit (Sun et al., 1992; D’Aoust et al., 1999). Invertases that catalyze the non-reversible cleavage of sucrose into fructose and glucose are present in three isoforms that all differ in their biochemical properties and subcellular localization. Soluble NI is located in the cytosol, soluble AI in the vacuole and insoluble AI is bound to the CWAI. Invertases have been shown to play a major role in flowering by determining floral sink strength (Bihmidine et al., 2013). AI mainly plays a role in cell osmoregulation and cell expansion (Balk and de Boer, 1999; Ranwala and Miller, 2008; Kutschera and Niklas, 2013). CWAI plays a key role in phloem unloading by converting sucrose into hexoses after sucrose is translocated from the phloem to the apoplast (Roitsch and González, 2004), enabling petals to increase their sink strength.

Translocation of photoassimilates depends on source supply and sink demand. During high sink activity, a high use of photoassimilates and enhanced phloem unloading rate lower the turgor of sink phloem and thereby increase mass flow. This stimulates phloem loading and lower accumulation of carbohydrates in source leaves (Ainsworth and Bush, 2011). Lower carbohydrate concentrations in source leaves will stimulate photosynthetic activity by releasing feedback inhibition and by up-regulation of genes for photosynthesis (Koch, 1996). On the other hand, when sink demand is low, carbohydrates accumulate in source leaves, down-regulating photosynthesis by suppressing photosynthetic gene expression (Paul and Foyer, 2001). The amount of sucrose available in source leaves for transport to sinks depends on photosynthetic activity (Lemoine et al., 2013). Suboptimal light conditions not only create a shortage of photoassimilate supply from the leaves, but also decrease photoassimilate transport as the expression of a sucrose transporter can be downregulated (Ishibashi et al., 2014).

In azalea (*Rhododendron simsii* hybrids), flower quality is highly dependent on the continuous development from closed flower buds to fully open flowers (OF). This developmental process is supported by a constant availability of carbohydrates. One important step before flower forcing is breaking the flower bud’s dormancy. This can be done by an artificial cold treatment at 7°C in the dark which significantly lowers carbohydrate reserves in the plants (Christiaens et al., 2015). Light conditions during forcing must allow adequate photosynthesis to restore carbohydrate reserves. The minimum light conditions for photosynthesis are cultivar dependent; an early flowering cultivar requires a DLI of 2.4 mol m^-2^ d^-1^, while a late flowering cultivar requires 1.7 mol m^-2^ d^-1^ (Christiaens et al., 2014). These minimum DLIs are not always present in greenhouses during wintertime and lower DLIs are considered as suboptimal light conditions. Post-production quality of flowering may also be limited by consumer environments with low light. Our objective was to investigate the effect of suboptimal light conditions during flowering in the greenhouse and in consumer environments on the source-sink metabolism of azalea and the quality of flowering. Using an early- and late-flowering cultivar, we determined the relative expression levels of *RsSUS* and the activity of enzymes (NI, AI, CWAI, SUS) involved in the sucrose metabolism during forcing. Furthermore, we quantified soluble carbohydrates and starch levels in leaves and flowers.

## Materials and Methods

### Plant Material and Experimental Setup

Two cultivars that differ in their natural flowering time and chilling requirements to break flower bud dormancy were used in this experiment. Four cuttings of the early flowering cultivar ‘Nordlicht’ or the late flowering cultivar ‘Sachsenstern’ were placed into the final pot (12 cm diameter) with a mixture of 9:1 peat:coconut fibers (v/v) (pH 4.5) and were covered with plastic to initiate rooting (December 2009) in a greenhouse. Soil temperature of 23–25°C was used to stimulate root formation without the use of rooting hormones. After 10 weeks (February 2010), the plastic foil was removed and plants were pruned at 2.5 cm to stimulate branching. Plants were pruned a second time (June 2010) to 7 cm and were transferred to an outdoor container field at the beginning of July 2010. During the vegetative growth phase, plants were watered automatically with 6-8 L m^-2^ based on irradiation sum (15 MJ m^-2^). Plants were fertilized with 0.5 kg/WM m^3^ Osmocote Exact Lo.Start (NPK 15-8-11 + 2MgO + TE, Everris) mixed with the substrate and extra fertigation (NPK 20-7-10, pH 4.5, EC 0.8-1.5 mS/cm). On August 3rd, 2010, plants were treated weekly with plant growth regulators (six applications with 2.25 g L^-1^ chlormequat) to initiate flower induction and suppress the outgrowth of axillary buds. When the style started to enlarge (flower bud stage 7; Bodson, 1983) in flower buds of ‘Nordlicht’ and when ovules were formed in the ovary (flower bud stage 7–8) for ‘Sachsenstern’, plants were moved to a dark cold room (7°C) for five (‘Nordlicht’) or seven (‘Sachsenstern’) weeks to break flower bud dormancy (**Table 1**). From August 3rd until cold treatment, ‘Nordlicht’ and ‘Sachsenstern’ plants received an average light sum of 795 ± 440 J cm^-2^ day^-1^ and 779 ± 420 J cm^-2^ day^-1^, respectively. Samples (described below) of leaves and flower buds were removed from plants before and after cold treatment for analyses of gene expression and concentrations of soluble carbohydrates and starch.

**Table 1:** Overview of the different treatments with start date, duration, day length, and mean DLI for *Rhododendron simsii* ‘Nordlicht’ and *R. simsii* ‘Sachsenstern’.

	‘Nordlicht’				‘Sachsenstern’			
		
Treatment	Start date	Duration (days)	Day length (h)	DLI (mol m^-2^ d^-1^)	Start date	Duration (days)	Day length (h)	DLI (mol m^-2^ d^-1^)
Cold	29/09/2010	35	0	0	13/10/2010	49	0	0
*F*	02/11/2010	65^3^	Natural (8.5)^2^	1.3 ± 0.8	30/11/2010	83^3^	Natural (8.1) ^2^	1.4 ± 1.1
*First week F ^1^*	*02/11/2010*	*7*	*Natural (9.5)^2^*	*2.5 ± 0.7*	*30/11/2010*	*7*	*Natural (8.2) ^2^*	*0.9 ± 0.4*
FA	02/11/2010	65^3^	16	5.2 ± 0.8	30/11/2010	63^3^	16	5.2 ± 0.8
FAL	26/11/2010	42^4^	18	0.7	29/12/2010	41^4^	18	0.7


*Cold treatment consisted of five (‘Nordlicht’) or seven (‘Sachsenstern’) weeks at 7°C in dark conditions. Light conditions during flowering were: forcing at natural light conditions (F), forcing at optimal light conditions with 16 h supplemental light (SON-T, 75–80 μmol m^-*2*^ s^-*1*^ at plant canopy level) (FA), and post-production flowering (FAL) where FA plants with CS were placed in a growth chamber under controlled conditions mimicking growing conditions similar to those in the consumer’s home (19.8 ± 0.4°C, 73.7 ± 9.5% RH, and 18 h light at 11 μmol m^-*2*^ s^-*1*^). ^1^First week F: this row provides information on the light conditions during the first week of forcing at natural light conditions. ^2^Average natural day length during the forcing period. ^3^The number of days between the start of forcing and the end of measurements to assess the quality of flowering. ^4^The number of days in the growth chamber until the end of measurements to assess the quality of flowering.*

After cold treatment, plants were split into two groups. One group (F) was forced in a greenhouse under natural light conditions and a second group (FA) had 16 h supplementary light (SON-T, 75-80 μmol m^-2^ s^-1^ at plant canopy level) (**Table 1**). Natural light conditions were considered as suboptimal light conditions because DLIs were lower than the minimum DLI for photosynthesis as determined by Christiaens et al. (2014). Greenhouse temperature during forcing was 21.2 ± 0.3°C and relative humidity was 57.8 ± 5.5%. During the six weeks of forcing, samples of leaves and flower buds were taken weekly, as described below, for gene expression analysis. In addition, during forcing samples were taken for analyses of carbohydrate concentrations (leaves and flower buds) and enzyme activity (petals from flower buds) at four developmental stages: after one week of forcing when buds were still closed (G), color-showing buds (CS), buds in the candle stage (CA), and fully OF.

To determine post-production quality, only plants forced with supplemental light (FA) were examined, as forcing under natural light conditions (F) resulted in poor flowering during greenhouse forcing. Twenty-four (‘Nordlicht’) or 29 (‘Sachsenstern’) days after the start of greenhouse forcing, half of the plants in the FA treatment, were placed in a growth chamber under controlled conditions that mimicked growing conditions similar to those in the consumer’s home (FAL) (19.8 ± 0.4°C, 73.7 ± 9.5% RH and 18 h light at 11 μmol m^-2^ s^-1^). Leaves and flower buds on FAL plants were sampled for gene expression, enzymes and carbohydrates as described above.

During vegetative growth outside, light intensity was measured with a solarimeter on the weather station located on the greenhouses and 20-minute means were registered by the climate computer (AEM/Mereg, Maasbree, the Netherlands). Photosynthetic active radiation during the forcing experiment (QS, Delta-T Devices, Cambridge, UK) was measured continuously in the greenhouse at canopy level (data were recorded every 5 min by a data logger (34970A, Agilent Technologies), and measured once in the growth chamber (constant light intensity) at canopy level.

### Gene Expression Analysis

A candidate gene for *RsSUS* (Acc. N° HG969196) was previously isolated; RT-qPCR primers are described in Christiaens et al. (2015). Leaf disks of two leaves (first mature leaf below the flower bud on two branches) per plant were harvested directly in Eppendorf tubes by using the lid to push out a 0.5 cm^2^ disk. All leaf samples from three plants per treatment were placed in one Eppendorf tube (six leaf disks per treatment). Flower bud samples (two per plant) were harvested and pooled as described above for leaf samples (six flower buds per treatment). All sampling was done at the end of the light period and samples were immediately frozen in liquid nitrogen. Plant material was stored at –80°C prior to analysis. RNA extraction, quality control, and RT-qPCR were done as described in Christiaens et al. (2015) and were done according to the MQE guidelines (Bustin et al., 2009) wherever possible. For every sample, two technical replicates were analyzed. For flower buds, a set of azalea reference genes (De Keyser et al., 2013) was validated; the geometric mean of RG5 and RG173 was used. For leaves, three reference genes were used according to Christiaens et al. (2015). Results of RNA quality control and run specific amplification efficiencies are presented as supplementary data (Supplementary Tables S1 and S2). A SPUD assay on similar leaf RNA samples confirmed no PCR inhibition was present in spite of low absorption ratios. No DNA contamination problems were reported from the noRT samples and melting profiles proved the absence of primer dimers.

### Enzyme Activities

All petals from flower buds were harvested from six plants per treatment; petals from two plants were bulked as one biological replicate (three biological replicates in total). Soluble AI, soluble NI, and cell wall-bound AI were analyzed in the petals. Petal SUS activity (sucrose cleavage direction) could not be detected in a consistent way, indicating activity at the edge of our detection limit. For the extraction of the soluble invertases, 2 g petal material was homogenized in 6 ml ice-cold extraction buffer (50 mM Hepes-NaOH, 20 mM MgCl_2_.6H_2_O, 5 mM dithiothreitol (DTT), 10 mM iso-ascorbic acid, 1 mM Na_2_EDTA, 0.1% (v/v) Triton X-100, pH 7.5) and filtered over Miracloth. The filtrate, which contains the soluble invertases, was centrifuged at 10,000 g at 4°C for 10 min. The residue with the cell wall-bound AI was washed three times with extraction buffer without DTT and Triton X-100 and incubated for 24 h in 3 ml incubation buffer (20 mM MES-KOH, 1 M NaCl, 5 mM DTT, pH 6). After incubation, samples were centrifuged at 10,000 *g* at 4°C for 10 min. To determine the activity of AI (soluble and cell-wall bound), 50 μl of the extract was incubated with 200 μl reaction buffer (100 mM acetate buffer, 100 mM sucrose, pH 5) for 45 min at 30°C (three technical replications for each extract). The reaction was stopped in boiling water; a blank was placed immediately into boiling water. The samples were cooled on ice and the glucose formed was determined by a LabAssay Glucose kit (Wako Chemicals GmbH, Neuss, Germany). The same procedure was followed to determine the activity of NI, but the reaction buffer contained 50 mM Hepes-NaOH and 50 mM sucrose at pH 7. Enzyme activities are expressed as μmol (glucose) min^-1^ g^-1^ (protein). To do so, protein content of the extracts was determined according to the method of Bradford (1976).

### Soluble Carbohydrates and Starch Content

Leaves (first mature leaves below flower buds) and flower buds/petals were harvested from six plants per treatment; tissues from two plants were bulked as one biological replicate (three biological replicates in total). Ground tissue (200 mg) of leaves and flower buds was extracted in 6 ml 80% ethanol for 3 h at 45°C. After centrifugation at 7,500 *g* (5 min), the supernatant was purified with 50 mg mL^-1^ PVPP. The concentrations of glucose, fructose, and sucrose in filtered (0.45 μm, Millipore) diluted samples were quantified by means of high performance anion-exchange chromatography with pulsed amperometric detection (HPAE-PAD) using a Dionex series chromatograph, equipped with a CarboPack PA10 column, a pulsed amperometric detector and a gold electrode.

The concentration of starch was determined by the acid hydrolysis of the remaining pellet after extraction of the soluble carbohydrates. The dried pellet was treated with 1 M HCl for 1 h at 95°C for starch hydrolysis. The pH of the supernatant was adjusted to 7.6 and the sample diluted to 10 mL. Starch content, expressed as glucose equivalents, was determined enzymatically by the reduction of NADP^+^ (measured at 340 nm, UV/VIS 916, GBC Scientific Equipment, Australia) with a hexokinase/glucose-6-phosphate dehydrogenase assay.

### Assessment of Flowering Quality

During the experiment, flowering of 10 plants per treatment was tracked by weekly counting of the number of buds in different developmental stages: green (closed) buds (G), CS, CA, and fully OF. Time of flowering was determined as the number of days between start of forcing and 10% CS. The total flowering percentage was calculated as the sum of % CS + % CA + % OF and used to determine the homogeneity of flowering as days between 10 and 90% flowering.

### Statistical Analysis

From a total of 46 plants per treatment, 10 plants were randomly chosen for flower assessment and the remaining plants were randomly used for sampling. All data were tested for normality with a Shapiro–Wilk test. The effect of the cold treatment on carbohydrate content was determined using the parametric Student’s *t*-test (equal variances shown by Levene’s test). Data on enzyme activity were analyzed with the non-parametric Kruskal–Wallis test. The effect of flower stage on carbohydrates during forcing was determined by means of the parametric analysis of variance (ANOVA) followed by a Tukey HSD test to separate means when variances were equal (Levene’s test) or by the ANOVA for unequal variances (Welch *F* test) followed by the Ryan–Einot–Gabriel–Welsch (REGW-F) *post hoc* test. The effect of light conditions on carbohydrates during forcing for each flower stage was determined by means of ANOVA for equal variances or the Welch *F* test for unequal variances; or a Student’s *t*-test or Welch *t*-test when only two light levels could be compared. The effect of light conditions on time of flowering, homogeneity of flowering and maximum %OF was analyzed by the non-parametric Mann–Whitney *U* test with Bonferroni correction (*p* < 0.002). A significance level of α = 0.05 was used for all analyses, except noted otherwise. Data were analyzed using SPSS statistical software Version 19.0 (SPSS Inc., Chicago, IL, USA).

## Results

### Effects of Cold Treatment on Carbohydrate Metabolism

For samples taken before and at the end of cold storage, normalization factor stability (*M*-value) within the leaf gene expression assays was 0.572 (CV = 0.224) and 0.425 (CV = 0.168) for ‘Nordlicht’ and ‘Sachsenstern’, respectively, and for flower bud assays 0.490 (CV = 0.171) and 0.212 (CV = 0.074) for ‘Nordlicht’ and ‘Sachsenstern’, respectively. In leaves of the cultivar ‘Nordlicht’, *RsSUS* expression increased twofold as a result of the cold treatment (**Table 2**), while there was no effect of cold treatment on leaf gene expression in ‘Sachsenstern’. Cold treatment did not result in a clear change in *RsSUS* flower bud gene expression in either cultivar.

**Table 2:** *RsSUS* relative gene expression levels (log10-transformed, means ± SE) and carbohydrate concentrations in leaves and flower buds before and after a cold treatment at 7°C in the dark for five weeks (*R. simsii* ‘Nordlicht’) and seven weeks (*R. simsii* ‘Sachsenstern’).

	‘Nordlicht’				‘Sachsenstern’			
		
	Leaf		Flower bud		Leaf		Flower bud	
				
Weeks at 7°C	0	5	0	5	0	7	0	7
*RsSUS*^1^	0.44 ± 0.03	1.02 ± 0.01	0.84 ± 0.02	0.66 ± 0.07	0.99 ± 0.04	1.06 ± 0.01	0.85 ± 0.02	1.22 ± 0.03
Glucose^2^ (mg g^-1^ FW)	2.11 b	4.51 a	0.64 b	1.97 a	1.75 a	1.35 a	1.06 a	0.91 a
Fructose^2^ (mg g^-1^ FW)	1.68 b	3.54 a	0.37 b	0.92 a	1.15 a	1.28 a	0.40 b	0.50 a
Sucrose^2^ (mg g^-1^ FW)	9.62 a	6.96 b	4.17 b	6.97 a	6.85 a	4.50 b	3.82 b	4.76 a
Starch^2^ (mg g^-1^ FW)	45.26 a	8.06 b	0.88 a	1.02 a	26.84 a	2.41 b	0.69 a	0.63 a


*Cold treatment started when the style started to enlarge in flower buds of ‘Nordlicht’ and when ovules were formed in the ovary (flower bud stage 7–8) for ‘Sachsenstern’. ^1^Means (*n* = 2) ± SE for *RsSUS* relative gene expression. ^2^ Means (*n* = 3) of glucose, fructose, sucrose, and starch concentrations in leaves or flower buds. Means within a cultivar and structure followed by different letters are significantly different (*p* = 0.05, Student’s *t*-test or Welch *t*-test) between the start and end of cold treatment.*

Concentrations of glucose and fructose in leaves and flower buds of ‘Nordlicht’ after cold storage were more than two times greater than before cold storage (**Table 2**). In contrast, cold storage had little influence on soluble sugar concentrations in leaves and flower buds of ‘Sachsenstern’. Effects of cold storage on sucrose and starch concentrations in leaves and flower buds were similar between the two cultivars. Cold storage decreased sucrose concentrations in leaves and increased sucrose concentrations in flower buds. Starch concentrations decreased in leaves during cold storage, but cold storage had no influence on starch concentrations in flower buds.

### *RsSUS* Gene Expression during Flowering

For samples taken during flowering, the *M*-value within leaf gene expression assays was 0.464 (CV = 0.186) and 0.465 (CV = 0.192) for ‘Nordlicht’ and ‘Sachsenstern’, respectively, and for flower bud assays 0.486 (CV = 0.168) and 0.413 (CV = 0.144) for ‘Nordlicht’ and ‘Sachsenstern’, respectively.

**FIGURE 1:**
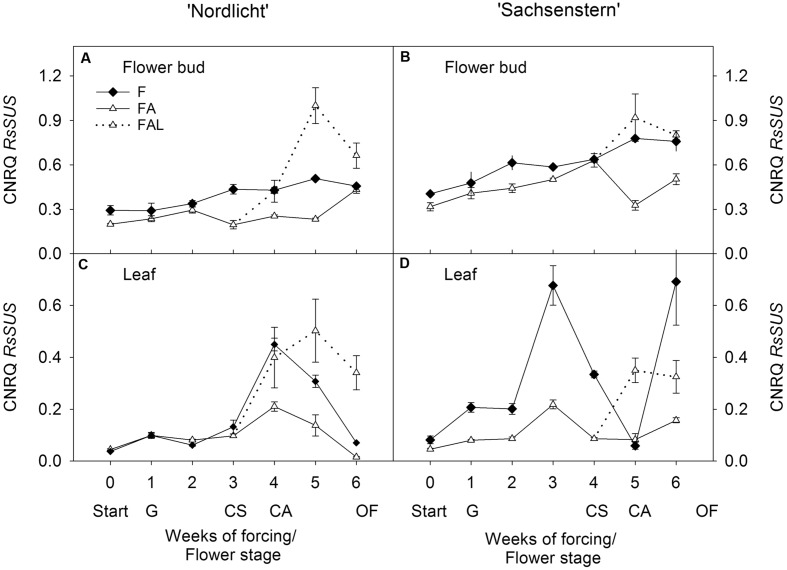
**Gene expression profiles (CNRQ, not log-transformed) of *RsSUS* in flower buds **(A)** and leaves **(C)** of *Rhododendron simsii* ‘Nordlicht’ and in flower buds **(B)** and leaves **(D)** of *R. simsii* ‘Sachsenstern’ during flowering in the greenhouse under natural light conditions (F), under optimal light conditions (FA) (natural light + 16 h supplementary light at 75–80 μmol m^-2^ s^-1^ at plant canopy level) and during flowering in the growth chamber (FAL) under controlled conditions mimicking growing conditions similar to those in the consumer’s home (19.8 ± 0.4°C, 73.7 ± 9.5% RH, and 18 h light at 11 μmol m^-2^ s^-1^) starting with FA plants with CS.** X-axis: weeks of forcing and flower developmental stage: Start: start of forcing, G: green buds after 1 week of forcing, CS: color-showing stage (at which half of the FA plants were transferred to FAL), CA: candle stage, OF: open flowers. Error bars indicate SE.

In both cultivars, light treatments during forcing in the greenhouse (F and FA) had no effect on *RsSUS* expression in flower buds (**Figures 1A,B**). In ‘Nordlicht’, leaf *RsSUS* expression increased after 3 weeks without supplemental light (F) and then decreased to the level of FA (**Figure 1C**). The F treatment caused similar changes in leaf *RsSUS* expression in ‘Sachsenstern’, although *RsSUS* expression fluctuated more, most likely due to differences in natural light conditions in the greenhouse at the time of sampling (**Figure 1D**). When both cultivars were transferred from the greenhouse with supplemental light (FA) to the growth chamber mimicking growing conditions similar to those of the consumer’s home (FAL), *RsSUS* expression increased, both in leaves and flower buds (**Figure 1**).

### Enzymatic Activity during Flowering

Invertase activity in F and FA flower buds after one week of forcing (G) was on the edge of the detection limit, for both cultivars (data not shown). During further forcing, the effect of light conditions on invertase activity (AI, NI, CWAI, and total invertase activity) (**Table 3**) was not statistically different for both cultivars. Total invertase activity did show a significant difference according to the Kruskal–Wallis test between flower stages (*p* = 0.001 for ‘Nordlicht’ and *p* = 0.005 for ‘Sachsenstern’). For ‘Sachsenstern’ this was due to significant differences in AI (*p* = 0.012) and NI (*p* = 0.007), and for ‘Nordlicht’ it was due to a significant difference in AI (*p* = 0.001). Total invertase activity in F and FA ‘Nordlicht’ and FA ‘Sachsenstern’ flower buds increased between CS and CAs. Sampling of F flowers of ‘Sachsenstern’ was not done at CA and OF because buds hardly developed further than the CS stage. Total invertase activity in ‘Nordlicht’ flowers was predominantly AI activity in all light conditions and all flower developmental stages. In CA and fully OFs of ‘Sachsenstern’ NI activity was highest. In CS of ‘Sachsenstern’ NI activity was nearly absent and CWAI was highest in the FA treatment, while in the F treatment both AI and CWAI were similar.

**Table 3:** Protein concentration, activity of AI, NI, cell-wall bound AI (CWAI), and total invertase (total I = AI + NI + CWAI) in developing flowers of *R. simsii* ‘Nordlicht’ and *R. simsii* ‘Sachsenstern’ at the color-showing stage (CS), candle stage (CA), and open flowers (OF) during flowering under different light conditions.

Cultivar	Flower stage	Light conditions^1^	Protein (μg g^-1^ FW)	AI	NI	CWAI	Total I
			(μg g^-1^ FW)	(μmol glucose min^-1^ g^-1^ (protein))			
‘Nordlicht’	CS	F	145 ± 30	202 ± 54	37 ± 37	178 ± 176	417 ± 194
		FA	178 ± 39	51 ± 27	11 ± 11	33 ± 32	95 ± 54
	CA	F	40 ± 8	807 ± 364	406 ± 225	51 ± 37	1264 ± 512
		FA	102 ± 4	701 ± 141	150 ± 150	0	851 ± 45
		FAL	76 ± 16	643 ± 216	208 ± 123	239 ± 127	1090 ± 185
	OF	F	156 ± 12	189 ± 29	18 ± 18	70 ± 36	274 ± 66
		FA	85 ± 9	524 ± 169	212 ± 212	216 ± 117	952 ± 400
		FAL	143 ± 11	292 ± 18	125 ± 74	160 ± 80	577 ± 50
‘Sachsenstern’	CS	F	653 ± 81	22 ± 12	2 ± 2	12 ± 12	36 ± 6
		FA	479 ± 60	19 ± 18	2 ± 1	82 ± 35	103 ± 54
	CA	FA	72 ± 13	284 ± 34	867 ± 552	435 ± 63	1587 ± 569
		FAL	40 ± 4	866 ± 457	923 ± 479	0	1789 ± 895
	OF	FA	44 ± 3	577 ± 401	1604 ± 345	597 ± 47	2777 ± 792
		FAL	46 ± 4	504 ± 259	613 ± 253	216 ± 215	1333 ± 381


*Means ± SE, *n* = 3. ^1^Light conditions during flowering were: forcing at natural light conditions (F), forcing at optimal light conditions with 16 h supplemental light (SON-T, 75–80 μmol m^-*2*^ s^-*1*^ at plant canopy level) (FA), and post-production flowering (FAL) where FA plants with CS were placed in a growth chamber under controlled conditions mimicking growing conditions similar to those in the consumer’s home (19.8 ± 0.4°C, 73.7 ± 9.5% RH and 18 h light at 11 μmol m^-*2*^ s^-*1*^).*

### Carbohydrate Concentrations during Flowering

**FIGURE 2:**
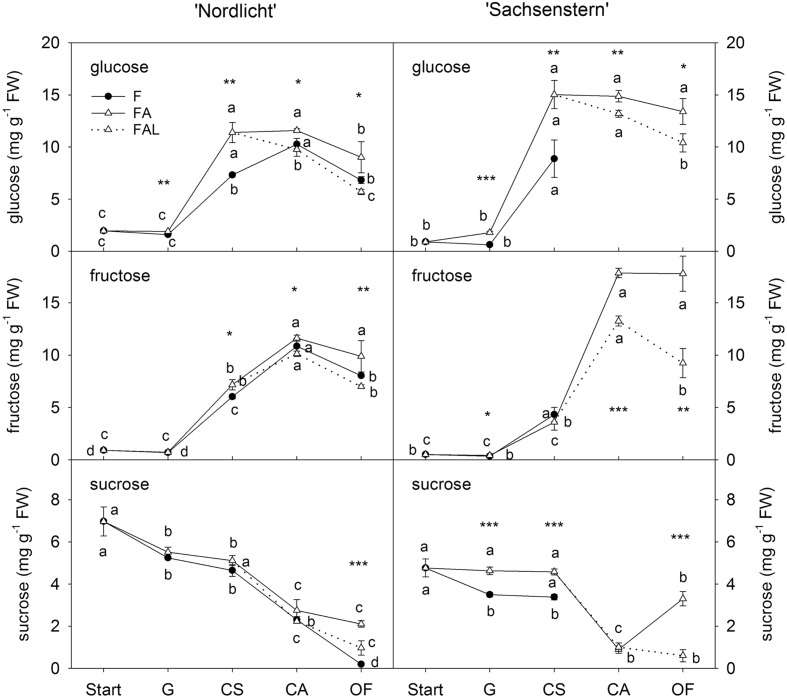
**Concentrations of glucose, fructose and sucrose in flower buds of *R. simsii* ‘Nordlicht’ and *R. simsii* ‘Sachsenstern’ during flowering in the greenhouse under natural light conditions (F), under optimal light conditions (FA) (natural light + 16 h supplementary light at 75–80 μmol m^-2^ s^-1^ at plant canopy level) and during flowering in the growth chamber (FAL) under controlled conditions mimicking growing conditions similar to those in the consumer’s home (19.8 ± 0.4°C, 73.7 ± 9.5% RH, and 18 h light at 11 μmol m^-2^ s^-1^) starting with FA plants with CS.** X-axis: Start: start of forcing, G: green buds after 1 week of forcing, CS: color-showing stage (at which half of the FA plants were transferred to FAL), CA: candle stage, OF: open flowers. Error bars indicate STDEV. Different letters indicate significant differences between the flower stages for the different light conditions (Tukey HSD or REGW-F, *p* = 0.05). Asterisk indicate significant differences between light conditions for each flower stage (^∗^*p* < 0.05, ^∗∗^*p* < 0.01, ^∗∗∗^*p* < 0.001, Student’s *t*-test, Welch *t*-test, Tukey HSD, or REGW-F).

Concentrations of glucose and fructose in flower buds of ‘Nordlicht’ and ‘Sachsenstern’ did not increase during the first week of forcing (from start to G) (**Figure 2**). A strong increase in glucose and fructose concentrations was seen between G and CA flowers for ‘Nordlicht’ and between G and CS flowers for ‘Sachsenstern’. This increase was higher in FA flowers compared to F flowers, except for fructose in ‘Sachsenstern’. In FA flowers, glucose and fructose levels stayed stable from CA to OF, except for glucose concentration in FA ‘Nordlicht’ flowers which decreased between CA and OF. Similarly glucose and fructose concentrations in F flowers of ‘Nordlicht’ decreased from CA to OF. Sampling of F flowers of ‘Sachsenstern’ was not done at CA and OF since buds hardly developed further than the CS stage. In contrast with flower glucose and fructose concentrations, flower sucrose concentrations decreased gradually in ‘Nordlicht’ from the start of forcing to OF. This decrease was similar for both forcing conditions (F and FA) up until CA flowers. From CA to OF, sucrose concentration in FA flowers stabilized, while in F flowers concentration dropped further. Flower bud sucrose concentrations in ‘Sachsenstern’ only showed a strong decrease from CS to CA in FA flowers, after which concentrations increased again from CA to OF.

**FIGURE 3:**
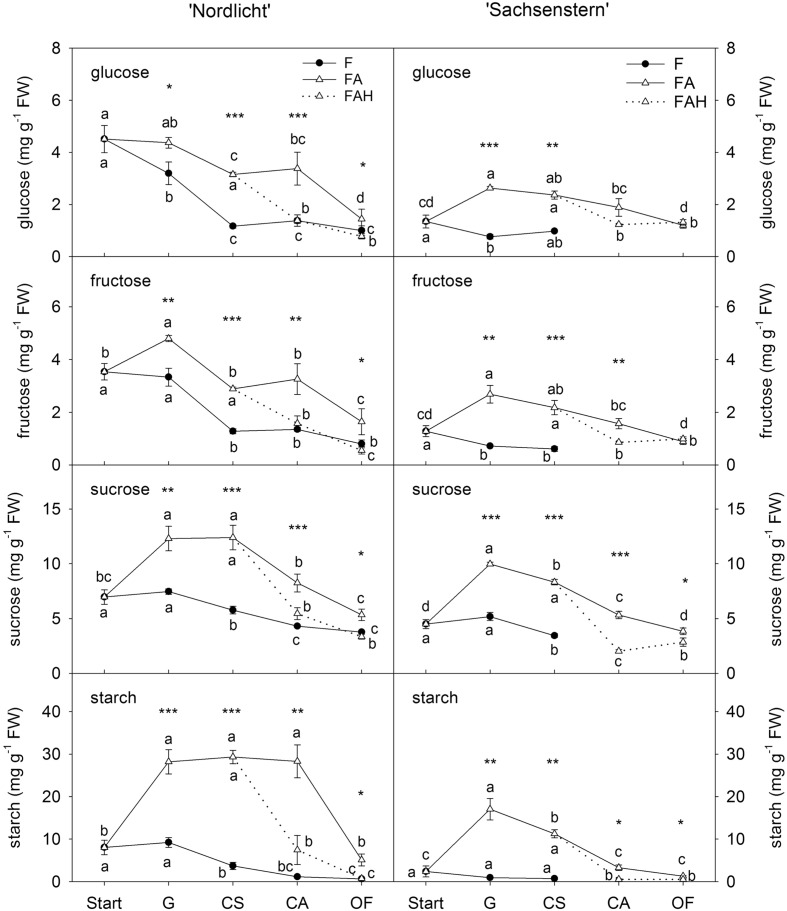
**Concentrations of glucose, fructose and sucrose in leaves of *R. simsii* ‘Nordlicht’ and *R. simsii* ‘Sachsenstern’ during flowering in the greenhouse under natural light conditions (F), under optimal light conditions (FA) (natural light + 16 h supplementary light at 75–80 μmol m^-2^ s^-1^ at plant canopy level) and during flowering in the growth chamber (FAL) under controlled conditions mimicking growing conditions similar to those in the consumer’s home (19.8 ± 0.4°C, 73.7 ± 9.5% RH, and 18 h light at 11 μmol m^-2^ s^-1^) starting with FA plants with CS.** X-axis: Start: start of forcing, G: green buds after 1 week of forcing, CS: color-showing stage (at which half of the FA plants were transferred to FAL), CA: candle stage, OF: open flowers. Error bars indicate STDEV. Different letters indicate significant differences between the flower stages for the different light conditions (Tukey HSD or REGW-F, *p* = 0.05). Asterisk indicate significant differences between light conditions for each flower stage (^∗^*p* < 0.05, ^∗∗^*p* < 0.01, ^∗∗∗^*p* < 0.001, Student’s *t*-test, Welch *t*-test, Tukey HSD, or REGW-F).

Leaf carbohydrates (**Figure 3**) showed opposite trends compared to flower carbohydrate concentrations during the development of a closed flower bud to full bloom. In general, forcing conditions (F, FA, FAL) had a greater influence on leaf carbohydrate concentrations than flower carbohydrate concentrations. Forcing under natural conditions (F) resulted in a decrease of all leaf carbohydrates from the start of forcing to full bloom (OF) for both cultivars. In contrast, during the first week of greenhouse forcing (from start to G) with supplemental light (FA), concentrations of all carbohydrates increased in leaves of ‘Nordlicht’ and ‘Sachsenstern’, except for leaf glucose concentration in ‘Nordlicht’, which stayed stable. During further flower development from G to OF, concentrations of leaf glucose and fructose in FA ‘Nordlicht’ and all carbohydrates in FA ‘Sachsenstern’ decreased. Only leaf sucrose levels stayed stable from G to CS and leaf starch levels from G to CA in FA ‘Nordlicht’, after which levels decreased to OF. This latter decrease in leaf starch concentrations for ‘Nordlicht’ resulted in a higher concentration (29.3 mg g^-1^ FW) when plants were transferred to the FAL treatment compared to leaf starch levels in ‘Sachsenstern’ (12.6 mg g^-1^ FW). The lowest concentrations of leaf carbohydrates were reached at an earlier flower bud stage in the F treatment compared to the FA treatment.

In both cultivars, the transfer from FA to growth conditions mimicking the consumer’s home (FAL) resulted in lower carbohydrate levels in both flowers (**Figure 2**) and leaves (**Figure 3**). A significant difference was seen at CA and OF for flower glucose and fructose levels and all leaf carbohydrates in both cultivars, except for leaf glucose and fructose in ‘Sachsenstern’. FAL flower sucrose concentrations were only significantly lower at OF compared to FA flowers for both cultivars.

### Quality of Flowering

Time of flowering was 5 and 12 days earlier in FA compared to F for ‘Nordlicht’ and ‘Sachsenstern’, respectively (**Table 4**). Forcing with supplemental light (FA) also significantly improved the maximum percentage of fully OFs (%OF) compared to atural light conditions (F) for both cultivars. During FAL, %OF was significantly reduced compared to FA conditions. Flower homogeneity could not be calculated for the F treatment, as 90% flowering was not reached during the forcing period. For ‘Sachsenstern’ only, the flower homogeneity was affected by the transfer to the growth chamber as the number of days is 7 higher for FAL compared to FA.

**Table 4:** Time of flowering (number of days between start of forcing and 10% CS), flower homogeneity (number of days between 10 and 90% total flowering) and maximum percentage of OFs (max. % OF)^1^ for *R. simsii* ‘Nordlicht’ and *R. simsii* ‘Sachsenstern’ under different light conditions.

	‘Nordlicht’			‘Sachsenstern’		
		
Light conditions^2^	Time of flowering (days)^3^	Homogeneity (days)^3^	Maximum % OF^3^	Time of flowering (days)^3^	Homogeneity (days)^3^	Maximum % OF^3^
F	23 b	–	49.8 b	38 b	–	14.5 c
FA	18 a	12 a	82.0 a	26 a	20 a	62.8 a
FAL		12 a	54.4 b		27 a	43.9 b


*^1^Maximum percentage of OFs is the percentage of OF at full bloom (before flowers started to wilt). ^2^Light conditions during flowering were: forcing at natural light conditions (F), forcing at optimal light conditions with 16 h supplemental light (SON-T, 75–80 μmol m^-*2*^ s^-*1*^ at plant canopy level) (FA), and post-production flowering (FAL) where FA plants with CS were placed in a growth chamber under controlled conditions mimicking growing conditions similar to those in the consumer’s home (19.8 ± 0.4°C, 73.7 ± 9.5% RH, and 18 h light at 11 μmol m^-*2*^ s^-*1*^). ^3^Means (*n* = 10) within a column followed by different letters are significantly different (*p* = 0.02, Mann–Whitney *U* Test).*

## Discussion

### Sucrose Metabolism during Flowering under Optimal Conditions

Flower forcing under optimal light conditions increased the glucose and fructose concentrations in petals. This has been observed in many other species such as petunia, *Ranunculus*, rose, lily, and tulips (Hachiya and Noguchi, 2008; Shahri and Tahir, 2011), where glucose and fructose serve as osmoticum to promote water influx and petal expansion. At the same time sucrose concentrations decreased in petals during flowering. This conversion from sucrose to glucose and fructose is catalyzed by invertases. The invertase activity increased most at the CA of flower development. This high increase in invertase activity might be related to a higher sink activity in the CA due to increased size and a higher respiration rate when petals start to expand (CA) (Lay-Yee et al., 1992). Invertase cleavage of sucrose is predominantly done by the AIs in ‘Nordlicht’ but NI activity is also present. Also in rose petals AI (soluble and cell wall-bound) activities are higher than NI activities and increase during flower development. Increasing SUS activity was also detected in rose petals, though its activity was weak compared to the invertase activity (Yamada et al., 2007; Kumar et al., 2008a; Horibe et al., 2013). We could not detect SUS activity despite the expression of *RsSUS* in the petals. Expression of *AtSUS3* in flowers has been shown in *Arabidopsis* (Bieniawska et al., 2007). Both *AtSUS3* and *RsSUS* are classified into the SUS II group (Christiaens et al., 2015). Nevertheless, the expression levels found did not result in high enzyme activities in azalea petals, indicating that invertase activities play a dominant role. Indeed, invertase cleavage of sucrose predominates in tissues where carbohydrates are catabolized for respiration and it has been associated with cell expansion (Winter and Huber, 2000). In contrast, SUS activity is the dominant activity in accumulating sinks (e.g., fruit) when the products of sucrose cleavage are used for biosynthesis of carbohydrate polymers like starch (Sun et al., 1992; Winter and Huber, 2000; Koch, 2004; Moscatello et al., 2011). In azalea petals, starch is not present, unlike flowers such as *Alstroemeria* (Collier, 1997), rose (Sood et al., 2006; Kumar et al., 2008b) and *Dendrobium* (Yap et al., 2008). In these flowers, the petal starch content increases during the first stages of flower opening, and is used again towards a fully OF to further increase the glucose and fructose content. It seems that azalea flowers solely depend on carbohydrate reallocation from their leaves for flower opening.

### Effect of Suboptimal Light Conditions during Forcing on Sucrose Metabolism

Cold treatment (before forcing) decreased leaf carbohydrate content greatly and increased leaf *RsSUS* expression (also described in detail in Christiaens et al., 2015). Forcing under optimal conditions maintained the expression levels of *RsSUS*, while under suboptimal conditions expression was increased after three weeks of forcing. In addition, low natural light conditions made it impossible for both cultivars to increase their leaf carbohydrates, an indication of inadequate photosynthesis. During the whole forcing period under natural light, DLIs for both cultivars were lower than their respective minimum DLIs for photosynthesis (Christiaens et al., 2014), which makes the supply of photoassimilates too limited to increase starch reserves. Also in rose, low light levels limit photosynthesis and the amount of photoassimilates is insufficient to meet the flower demands (Mattson et al., 2008). In contrast, plants forced with supplemental light are able to build up some of their starch reserves during the first week of forcing, but the obtained levels were still much lower than before cold treatment.

Even though source supply was limited under natural light conditions, sink demand will be high in opening buds. Forcing conditions had no impact on the expression levels of *RsSUS* in flower buds. There was no statistical evidence for a higher total invertase activity in petals under suboptimal light conditions compared to optimal light conditions. Nevertheless, mean values tended to be higher during the first stages of flower opening, an effect also seen during shading of Japanese pear, where the activities of AI in the bud increase to enhance the sink strength under low light conditions (Ito et al., 2003). This increased sink strength might have stimulated the loading of sucrose at the source leaves to still provide high levels of carbohydrates to opening flowers. Indeed, the differences seen in soluble carbohydrates in the flowers of plants under optimal and suboptimal conditions were small. Because not all flower buds on plants under natural light developed, samples were taken from developing flowers. Probably the limited leaf carbohydrate pool was used for those buds that opened, indicating a strong inter-flower bud competition for assimilates. In cut flowers, longevity of OFs could be increased by removing other floral buds which compete for the inadequate amount of carbohydrates (van Doorn and Han, 2011). A similar effect in azalea is possible because the non-developing flower buds aborted and were thus excluded from carbohydrate supply, ensuring enough sugars provision to the flowers that do develop.

The overall result, however, is very poor flowering during forcing under natural light conditions. For ‘Sachsenstern’ only a few buds opened and no further analyses after the CS stage could be done. In contrast, ‘Nordlicht’ did have more buds that fully opened, but the percentage was still low. The difference between both cultivars might be found in the different carbon content at the start of forcing, which was markedly lower for ‘Sachsenstern’. Furthermore, the light conditions during the first week of forcing (**Table 1**) differed greatly. For ‘Nordlicht’ a DLI of 2.5 mol m^-2^ d^-1^ was measured, which is slightly higher than the minimum DLI of 2.1 mol m^-2^ d^-1^ and might have allowed production of photoassimilates. For ‘Sachsenstern’ the 0.9 mol m^-2^ d^-1^ was substantially lower than the minimum DLI of 1.7 mol m^-2^ d^-1^, indicating inadequate photosynthesis which is reflected in very low levels of carbohydrates in leaves.

### Post-Production Sucrose Metabolism and Quality of Flowering

The quality of flowering at the consumer’s home will be strongly influenced by the available leaf carbohydrates. When plants are transferred at the CS stage from optimal greenhouse forcing to a growth chamber mimicking growing conditions similar to the consumer’s home, an immediate drop in leaf carbohydrates and increase in leaf *RsSUS* expression will occur. Differences in the flower buds were much smaller but still lower levels of carbohydrates were measured. The expression of *RsSUS* in petals increased slightly, indicating a more important role in sink strength to attract carbohydrates from the leaves in low light conditions. Nevertheless, total invertase activity in flowers tended to decrease at full bloom in a post-production environment. In roses, post-production enzyme activities in flowers decreased (Horibe et al., 2013). In contrast with roses, our results show that for post-production flower opening of azalea, soluble carbohydrates in the petals are a result of the breakdown of starch in the leaves while post-production flower opening in cut roses relies on starch reserves in the petals (Kumar et al., 2007). Post-production quality of flowering is also markedly lower compared to flowering in optimal greenhouse forcing, indicating that petal starch reserves at the start at the post-production phase are still insufficient to meet the petal carbohydrate demand.

## Author Contributions

AC, EDK, EP, JDR, BG, and M-CVL contributed to the conception and design of the work. AC and EDK carried out laboratory, experimental and/or statistical analyses. AC and EDK drafted the manuscript that was critically revised and approved by EP, JDR, BG, and M-CVL.

## Conflict of Interest Statement

The authors declare that the research was conducted in the absence of any commercial or financial relationships that could be construed as a potential conflict of interest.

## Abbreviations

AI: acid invertase
CWAI: cell wall bound AI
DLI: daily light integral
NI: neutral invertase
SPS: sucrose phosphate synthase, SUS, sucrose synthase
