# Supplementary material for: Suboptimal Light Conditions Influence Source-Sink Metabolism during Flowering
Source: Front Plant Sci. 2016 Mar 3;7:249. doi: 10.3389/fpls.2016.00249 (PMC4776122; doi:10.3389/fpls.2016.00249)
Supplement: Supplementary file 1 [file Table_1.DOCX]

Supplementary Material

Suboptimal light conditions influence source-sink metabolism during flowering

Annelies Christiaens1,2*, Ellen De Keyser3, Els Pauwels2, Jan De Riek3, Bruno Gobin2, Marie-Christine Van Labeke1*

*** Correspondence:** Annelies Christiaens, PCS Ornamental Plant Research, Schaessestraat 18, Destelbergen, 9080, Belgium. annelies.christiaens@pcsierteelt.be

Marie-Christine Van Labeke, Department of Plant Production, Faculty of Bioscience Engineering, Ghent University, Coupure Links 653, Ghent, 9000, Belgium. mariechristine.vanlabeke@ugent.be

**Supplementary Table 1.** RNA quantity and purity (A260/A280 and A260/A230) was measured of each sample using a NanoDrop spectrophotometer.

| **Sample ID** | **Weeks of forcing** | **Treatment** | **Genotype** | **Sample type** | **Concentration (ng/µl)** | **A_260_/A_280_** | **A_260_/A_230_** |
| --- | --- | --- | --- | --- | --- | --- | --- |
| HV87 | 0 | F | Nordlicht | flower bud | 743,47 | 2,07 | 2,4 |
| HV159 | 1 | F | Nordlicht | flower bud | 607,39 | 2,02 | 2,42 |
| HV255 | 2 | F | Nordlicht | flower bud | 718,92 | 2,05 | 2,47 |
| HV327 | 3 | F | Nordlicht | flower bud | 694,42 | 2,04 | 2,37 |
| HV399 | 4 | F | Nordlicht | flower bud | 850,35 | 2,09 | 2,43 |
| HV471 | 5 | F | Nordlicht | flower bud | 776,98 | 2,08 | 2,39 |
| HV543 | 6 | F | Nordlicht | flower bud | 742,67 | 2,06 | 2,37 |
| HV95 | 0 | FA | Nordlicht | flower bud | 1514,64 | 2,04 | 2,25 |
| HV167 | 1 | FA | Nordlicht | flower bud | 1712,49 | 2,02 | 2,24 |
| HV263 | 2 | FA | Nordlicht | flower bud | 1303,02 | 2,01 | 2,29 |
| HV335 | 3 | FA | Nordlicht | flower bud | 1377,19 | 2,04 | 2,26 |
| HV407 | 4 | FA | Nordlicht | flower bud | 882,25 | 2,08 | 2,37 |
| HV480 | 5 | FA | Nordlicht | flower bud | 371,41 | 2 | 2,37 |
| HV559 | 6 | FA | Nordlicht | flower bud | 448,7 | 1,97 | 2,27 |
| HV415 | 4 | FAL | Nordlicht | flower bud | 814,69 | 2,05 | 2,35 |
| HV488 | 5 | FAL | Nordlicht | flower bud | 358,65 | 1,96 | 2,39 |
| HV567 | 6 | FAL | Nordlicht | flower bud | 745,52 | 2,04 | 2,4 |
| HV86 | 0 | F | Nordlicht | leaf | 191,81 | 1,81 | 0,9 |
| HV157 | 1 | F | Nordlicht | leaf | 136,49 | 1,96 | 1,32 |
| HV253 | 2 | F | Nordlicht | leaf | 169,42 | 1,83 | 0,95 |
| HV325 | 3 | F | Nordlicht | leaf | 195,06 | 1,82 | 0,89 |
| HV397 | 4 | F | Nordlicht | leaf | 161,21 | 1,9 | 1,11 |
| HV469 | 5 | F | Nordlicht | leaf | 141,79 | 1,86 | 1,01 |
| HV541 | 6 | F | Nordlicht | leaf | 212,12 | 1,97 | 1,51 |
| HV93 | 0 | FA | Nordlicht | leaf | 109,79 | 1,74 | 0,8 |
| HV165 | 1 | FA | Nordlicht | leaf | 105,99 | 1,74 | 0,81 |
| HV261 | 2 | FA | Nordlicht | leaf | 133,66 | 1,81 | 0,9 |
| HV333 | 3 | FA | Nordlicht | leaf | 118,59 | 1,78 | 0,92 |
| HV405 | 4 | FA | Nordlicht | leaf | 116,61 | 1,78 | 0,88 |
| HV477 | 5 | FA | Nordlicht | leaf | 118,91 | 1,75 | 0,82 |
| HV557 | 6 | FA | Nordlicht | leaf | 236,61 | 1,97 | 2,06 |
| HV413 | 4 | FAL | Nordlicht | leaf | 109,72 | 1,73 | 0,75 |
| HV485 | 5 | FAL | Nordlicht | leaf | 122,76 | 1,77 | 0,91 |
| HV565 | 6 | FAL | Nordlicht | leaf | 109,05 | 1,72 | 0,8 |
| S71 | 0 | F | Sachsenstern | flower bud | 874,68 | 2,07 | 2,45 |
| S119 | 1 | F | Sachsenstern | flower bud | 798,12 | 2,03 | 2,44 |
| S167 | 2 | F | Sachsenstern | flower bud | 804,76 | 2,05 | 2,46 |
| S251 | 3 | F | Sachsenstern | flower bud | 742,94 | 2,04 | 2,43 |
| S283 | 4 | F | Sachsenstern | flower bud | 909,14 | 2,04 | 2,48 |
| S347 | 5 | F | Sachsenstern | flower bud | 691,46 | 2,08 | 2,45 |
| S419 | 6 | F | Sachsenstern | flower bud | 652,91 | 2,02 | 2,48 |
| S79 | 0 | FA | Sachsenstern | flower bud | 709,13 | 2,03 | 2,49 |
| S127 | 1 | FA | Sachsenstern | flower bud | 803,94 | 2,04 | 2,94 |
| S175 | 2 | FA | Sachsenstern | flower bud | 549 | 2,07 | 2,5 |
| S259 | 3 | FA | Sachsenstern | flower bud | 769,62 | 2,04 | 2,42 |
| S291 | 4 | FA | Sachsenstern | flower bud | 782,46 | 2,01 | 2,47 |
| S355 | 5 | FA | Sachsenstern | flower bud | 620,44 | 2 | 2,43 |
| S427 | 6 | FA | Sachsenstern | flower bud | 739,21 | 2,06 | 2,36 |
| S367 | 5 | FAL | Sachsenstern | flower bud | 858,63 | 2,04 | 2,42 |
| S435 | 6 | FAL | Sachsenstern | flower bud | 839,86 | 2,06 | 2,41 |
| S69 | 0 | F | Sachsenstern | leaf | 114,68 | 1,94 | 0,98 |
| S117 | 1 | F | Sachsenstern | leaf | 201,66 | 1,7 | 0,62 |
| S165 | 2 | F | Sachsenstern | leaf | 191,09 | 2 | 1,4 |
| S249 | 3 | F | Sachsenstern | leaf | 302,56 | 1,56 | 0,74 |
| S281 | 4 | F | Sachsenstern | leaf | 235,59 | 1,63 | 0,67 |
| S345 | 5 | F | Sachsenstern | leaf | 238,01 | 1,57 | 0,68 |
| S417 | 6 | F | Sachsenstern | leaf | 217,21 | 1,64 | 0,67 |
| S77 | 0 | FA | Sachsenstern | leaf | 180,55 | 1,55 | 0,67 |
| S125 | 1 | FA | Sachsenstern | leaf | 179,2 | 1,72 | 0,71 |
| S173 | 2 | FA | Sachsenstern | leaf | 237,87 | 1,87 | 1 |
| S257 | 3 | FA | Sachsenstern | leaf | 234,08 | 1,55 | 0,69 |
| S289 | 4 | FA | Sachsenstern | leaf | 262,81 | 1,54 | 0,73 |
| S353 | 5 | FA | Sachsenstern | leaf | 233,31 | 1,54 | 0,66 |
| S425 | 6 | FA | Sachsenstern | leaf | 143,42 | 1,57 | 0,56 |
| S365 | 5 | FAL | Sachsenstern | leaf | 220,56 | 1,53 | 0,62 |
| S433 | 6 | FAL | Sachsenstern | leaf | 220,29 | 1,53 | 0,64 |
