# Supplementary material for: Suboptimal Light Conditions Influence Source-Sink Metabolism during Flowering
Source: Front Plant Sci. 2016 Mar 3;7:249. doi: 10.3389/fpls.2016.00249 (PMC4776122; doi:10.3389/fpls.2016.00249)
Supplement: Supplementary file 2 [file Table_2.DOCX]

Supplementary Material

Suboptimal light conditions influence source-sink metabolism during flowering

Annelies Christiaens1,2*, Ellen De Keyser3, Els Pauwels2, Jan De Riek3, Bruno Gobin2, Marie-Christine Van Labeke1*

*** Correspondence:** Annelies Christiaens, PCS Ornamental Plant Research, Schaessestraat 18, Destelbergen, 9080, Belgium. annelies.christiaens@pcsierteelt.be

Marie-Christine Van Labeke, Department of Plant Production, Faculty of Bioscience Engineering, Ghent University, Coupure Links 653, Ghent, 9000, Belgium. mariechristine.vanlabeke@ugent.be

**Supplementary Table 2.** Summary of slopes, intercepts and R² of the standard curves made of a dilution of 5/6 standards for each gene under analysis. The PCR efficiciency (E) and the standard deviation on E (SD(E)) were calculated according to the formulas described in Hellemans et al. (2007). Data were calculated for every experiment.

| **Gene** | **Type** | **Genotype** | **Sample Type** | **slope** | **intercept** | **R²** | **# of standards** | **E** | **SD(E)** |
| --- | --- | --- | --- | --- | --- | --- | --- | --- | --- |
| RsSUS | target gene | Nordlicht | Leaf | -3,672 | 38,807 | 0,999 | 6 | 1,872 | 0,009 |
| GAPDH | reference gene | Nordlicht | Leaf | -3,688 | 40,574 | 0,999 | 6 | 1,867 | 0,013 |
| RG5 | reference gene | Nordlicht | Leaf | -3,484 | 33,621 | 0,999 | 6 | 1,936 | 0,013 |
| RG47 | reference gene | Nordlicht | Leaf | -3,766 | 42,492 | 0,999 | 5 | 1,843 | 0,015 |
| RsSUS | target gene | Nordlicht | Flower bud | -3,634 | 38,565 | 0,999 | 6 | 1,884 | 0,011 |
| RG5 | reference gene | Nordlicht | Flower bud | -3,479 | 33,424 | 0,999 | 6 | 1,938 | 0,014 |
| RG173 | reference gene | Nordlicht | Flower bud | -3,639 | 38,392 | 0,999 | 6 | 1,883 | 0,014 |
| RsSUS | target gene | Sachsenstern | Leaf | -3,718 | 39,028 | 0,999 | 6 | 1,857 | 0,012 |
| GAPDH | reference gene | Sachsenstern | Leaf | -3,741 | 40,825 | 0,998 | 6 | 1,85 | 0,015 |
| RG5 | reference gene | Sachsenstern | Leaf | -3,484 | 33,621 | 0,999 | 6 | 1,936 | 0,013 |
| RG47 | reference gene | Sachsenstern | Leaf | -3,765 | 42,492 | 0,999 | 5 | 1,843 | 0,015 |
| RsSUS | target gene | Sachsenstern | Flower bud | -3,634 | 38,565 | 0,999 | 6 | 1,884 | 0,011 |
| RG5 | reference gene | Sachsenstern | Flower bud | -3,504 | 33,648 | 1 | 6 | 1,929 | 0,006 |
| RG173 | reference gene | Sachsenstern | Flower bud | -3,639 | 38,392 | 0,999 | 6 | 1,883 | 0,014 |

Hellemans, J., Mortier, G., De Paepe, A., Speleman, F., Vandesompele, J. (2007). qBase relative quantification framework and software for management and auto- mated analysis of real-time quantitative PCR data. Genome Biology, 8, R19.
